# Up-regulation of long non-coding RNA-PCAT-1 promotes invasion and metastasis in esophageal squamous cell carcinoma

**DOI:** 10.17179/excli2018-1847

**Published:** 2019-06-17

**Authors:** Maryam Razavi, Saeid Ghorbian

**Affiliations:** 1 Department of Molecular Genetics, Ahar Branch, Islamic Azad University, Ahar, Iran

**Keywords:** LncRNA-PCAT-1, esophageal squamous cell carcinoma, ESCC, lncRNA

## Abstract

Long non-coding RNA prostate cancer associated transcript-1 ncRNA (lncRNA-PCAT-1) plays an important role in the progression of prostate cancer. The present investigation was aimed to evaluate the potential roles of the *lncRNA-PCAT-1* gene expression changes in esophageal squamous cell carcinoma (ESCC) between Iranian population. In the case-control investigation, we have analyzed a total of 150 fresh tissue samples, compromising of 75 cancerous tissues and 75 adjacent normal tissues from patients with ESCC. We used quantitative Real-time polymerase chain reaction (qRT-PCR) to evaluate the *lncRNA-PCAT-1* gene expression levels in ESCC patients and correlation between the *lncRNA-PCAT-1* expression changes and clinical characteristics. Our findings showed that the *lncRNA-PCAT-1* gene was up-regulated in cancerous tissues compared with the adjacent non-cancerous tissues (p=0.0016). In addition, the results revealed a significant correlation between up-regulating of *lncRNA-PCAT-1* and hot liquid drinking (p =0.017). These findings offer the potential roles of *lncRNA-PCAT-1* in the pathogenesis of ESCC and may consider as a candidate prognostic biomarker for ESCC in an Iranian population.

## Introduction

Newer studies have revealed that thousands of long non-coding RNAs (lncRNAs), characterized as transcripts bigger than two hundred nucleotides length, do not encode protein, expressed in tissue-specific patterns and play crucial roles in aspects of several biological pathways (Bracken and Helin, 2009[2]; Faghihi and Wahlestedt, 2009[3]). High-throughput sequencing of RNA shows that the lncRNAs may influence in several cellular pathways such as cell cycle regulation, transcriptional gene silencing by cooperation with the chromatin-modifying proteins, histone modification, RNA maturation, and transport of proteins synthesized (Whitehead et al., 2009[22]; Wang and Chang, 2011[20]). To date, the findings revealed that the lncRNAs involve in most of the mammalian genome, while a few functional lncRNAs have been characterized (Wapinski and Chang, 2011[21]). Investigations have been revealed that a significant association between deregulation of lncRNAs and cancerogenesis, such as colorectal, breast, lung, and hepatocellular carcinoma. LncRNAs can act as tumor suppressors or oncogenes, which are down-regulated or up-regulated in different tumors (Aalijahan and Ghorbian, 2019[1]; Shams and Ghorbian, 2019[14]; Sadeghpour and Ghorbian, 2019[12]; Lu et al., 2013[8]). Hence, further attention has been engaged to display the molecular mechanisms of lncRNAs in progress of cancers. Previously, PCAT-1 was characterized as a practical non-coding RNA particle, which was involved in progression of the subjects with prostate cancer. From the molecular viewpoint, it is determined that PCAT-1 was up-regulated in a subset of metastatic and high-grade localized prostate cancer (St Laurent et al., 2012[16]; Ponting et al., 2009[9]). Noteworthy, it has been confirmed that lncRNA-PCAT-1 also may contribute to cell proliferation in other types of carcinomas. Up to date, some investigators revealed that up-regulation of lncRNA-PCAT-1 was identified in advanced clinical stage and poor prognosis of colorectal, hepatocellular, and bladder cancers (Volders et al., 2013[19]; Struhl, 2007[17]; Schmitt and Chang, 2016[13]). Recently, some studies showed that up-regulation of lncRNA-PCAT-1 in a subgroup of aggressive cancers could play as a predictive biomarker for tumor detection and medication (Tseng et al., 2014[18]). However, the reliable association between lncRNA-PCAT-1 expression and progress and advancement of other neoplasia remains obscure. Within this situation, the ongoing survey was planned to analyze the gene expression pattern of lncRNA-PCAT-1 in the subject with ESCC and consider the relationship between lncRNA-PCAT-1 expression and clinical characteristics in an Iranian population.

## Materials and Methods

### Patients and samples

In the current case-control study, we have analyzed a total of 150 fresh tissue samples consisting of 75 cancerous tissues and 75 adjacent non-cancerous tissues from patients with ESCC, which were referred to the Tabriz International Hospital during September 2014 to November 2017. All samples were collected after endoscopy, diagnosed and confirmed by histopathological surveys.

For all cases, demographic information such as age, sex, clinical condition, and family history of esophageal cancer was indicated. Individuals who underwent chemotherapy, radiotherapy or proposed therapy were excluded. The sample was obtained according to protocols confirmed by the ethics committee of the Tabriz University of Medical Sciences and signed informed consent and questionnaire were received from each case. After sampling, all tissue specimens were instantaneously transferred to the microtubule containing RNA, later solution and stored at −80 °C until total RNA extraction. 

### RNA isolation and complementary DNA synthesis 

RNA extraction was carried out by the TRIzol reagent (Invitrogen) following the manufacturer's instructions. Quality and quantity of the extracted RNAs were evaluated by standard gel electrophoresis with 1 % agarose and UV spectrophotometry at 260/280 nm (using the NanoDrop^TM^ ND-1,000, NanoDrop Technology, Wilmington, DE, USA), respectively. The total RNA was reverse transcribed into cDNA using PrimeScript RT-PCR Kit (Takara, Japan) according to the manufacturer's protocols. The cDNA was synthesized by reverse transcription from 200 ng of RNA in a 20 µl solution containing 1 µl dNTP mixture (10 mM each), 4 µl 5×PrimeScript Buffer, 0.5 µl RNase Inhibitor, 1 µl random hexamer primer, 0.5 µl PrimeScriptRTase. cDNA synthesis was performed at 42 °C for 30 min, and the reaction was stopped by heating at 95 °C for 5 minutes.

### Quantitative real-time PCR 

The *lncRNA-PCAT-1* gene expression changes was evaluated by quantitative real-time polymerase chain reaction (q-real-time PCR), which was performed with 2.0 μl of cDNA, 1.0 μl of each primer and 10 μl of SYBR green Real-time PCR Master Mix (TaKaRa, Japan). QRT-PCR was performed using the ABI7500 System (Applied Biosystems, CA, USA). A total of 35 amplification cycles were performed at 94 °C for 15 seconds and 58 °C for 30 seconds. The gene encoding human *β-actin* was amplified as an internal control to verify equal loading of cDNA. The forward primer sequences used for *lncRNA-PCAT-1* gene was, 5´-TTGTGGAAGCCCCGCAAGGCCTGAA-3´ and reverse, 5´-TGTGGGGCCTGCACTGGCACTT-3´ (Shi et al., 2015[15]).

*β-actin* gene was also included as an internal control with primer sequences, forward: 5´-AAGGCCAACCGCGAGAAG-3´, reverse 5´-ACAGCCTGGATAGCAACGTACA-3´. All experimental assays were performed in triplicate. The relative amount of *lncRNA-PCAT-1* to *β-actin* was calculated using equation 2^−^^△△^^Ct^. 

### Statistical analysis

All statistical analyses were done using the SPSS version 22.0 (IBM Corp., Armonk, USA). The gene expression levels of *lncRNA-PCAT-1* in cancerous tissues and non-cancerous tissues were measured using the two-sided Student's t-test. The associations of *lncRNA-PCAT-1* gene expression with clinical features were evaluated by chi-square test. A two-tailed *p*<0.05 was considered to indicate a significant difference. 

## Results

From a total of 75 patients with confirmed ESCC, 47 patients (62.6 %) were male and 28 patients (37.4 %) were female. We calculated the mean age of 64.7±2.023 years (ranging from 39 to 66 years). 

**Figure 1:**
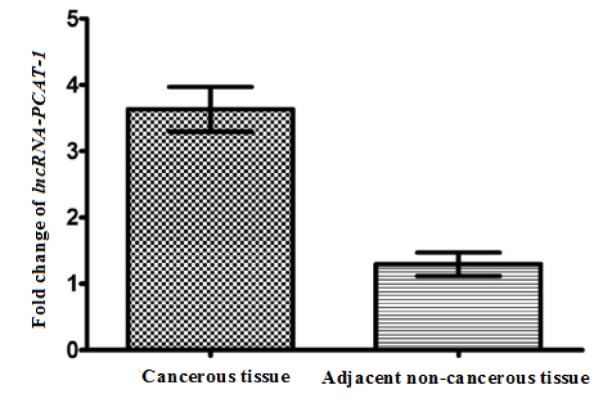
The expression level of *lncRNA-PCAT-1 *gene in cancerous tissues and adjacent non-cancerous tissues

Our findings revealed that the *lncRNA-PCAT-1* gene expression was up-regulated in ESCC patients (Figure 1(Fig. 1)). The *lncRNA-PCAT-1* gene expression change was measured using real-time PCR in 75 paired tissues of ESCC. Our findings revealed the *lncRNA-PCAT-1* was up-regulated in cancerous tissues compared with the adjacent non-cancerous tissues (p<0.0016) (Figure 1(Fig. 1)). We divided patients into high and low expression levels groups by a cut-off value determined based on the median cancerous/adjacent non-cancerous tissue ratio. The median amount of relative *lncRNA-PCAT-1* expression was 2.49 conforming to interpreting QRT-PCR in 75 specimens of ESCC. We calculated the median amount ≥2.49 of *lncRNA-PCAT-1* gene expression level was considered as a high-expression group and a median amount <2.49 selected as a low-expression group. Our evaluation revealed that the *lncRNA-PCAT-1* gene expression change in ESCC was correlated with the hot liquid drinking (p=0.017) (Table 1(Tab. 1)). Moreover, the findings did not show a positive correlation between *lncRNA-PCAT-1* gene expression change and other clinical features such as lymph node metastasis, age, alcohol drinking, sexuality, tumor stage, smoking status, and socioeconomic status (Table 1(Tab. 1)). 

## Discussion

Up to date, various lncRNA molecules have been demonstrated to contribute to tumor development and progression in many cancer types, which can be used as a powerful diagnostic and prognostic biomarker for the diseases (Volders et al., 2013[19]; Struhl, 2007[17]). Increasing evidence reveals some lncRNAs with various types of malignancies. Several investigators declared that the lncRNA molecules can perform compound processes on many crucial cancer phenotypes through their interactions with other cellular macromolecules consisting of DNA, protein, and RNA (Schmitt and Chang, 2016[13]). Accordingly, it has been indicated that lncRNA-PVT1, lncRNA-PCAT-1, lncRNA-CCAT2, lncRNA-PTCSC3, and lncRNA-HULC are associated with colorectal, prostate, thyroid, and hepatocellular cancers, respectively (Tseng et al., 2014[18]; Shi et al., 2015[15]; Ling et al., 2013[5]; Jendrzejewski et al., 2012[4]; Liu et al., 2012[7]). Some investigations were conducted on prostate-specific lncRNA-PCAT-1 as a novel prostate-specific regulator of cell proliferation and prostate cancer biomarker, which was found in the chromosome 8q24 located upstream of the myc gene (Shi et al., 2015[15]). 

In our survey, findings revealed that the lncRNA-PCAT-1 was overxpressed in ESCC cancerous tissues. Similarity to our results, Prensner et al. (2013[10]) declared that the lncRNA-PCAT-1 was significantly overexpressed in a subset of prostate cancer, predominantly metastases, and can contribute to the cell proliferation as a tumorigenesis factor (Prensner et al., 2013[10]). Moreover, the results indicated that the lncRNA-PCAT-1 deregulation was correlated with a subgroup of acute types of prostate malignancies, especially metastases (Shi et al., 2015[15]). 

Based on previous data, the lncRNA-PCAT-1 has also been overexpressed in other cancerous tumors such as lung, colorectal, gastric and hepatocellular carcinoma (Zhao et al., 2015[25]; Liu et al., 2015[6]; Yan et al., 2015[23]; Zhang et al., 2017[24]; Ren et al., 2017[11]). 

Zhao et al. (2015[25]) reported that overexpression of lncRNA-PCAT-1 may stimulate proliferation and progression in non-small cell lung cancer cells (NSCLC). The findings recommended the lncRNA-PCAT-1 act as an oncogenic in NSCLC progression and may be applicable as a novel curative approach for lung cancer (Zhao et al., 2015[25]). 

Liu et al. (2015[6]) showed that the lncRNA-PCAT-1 was down-regulated in bladder malignancy due to suppressing of lncRNA-PCAT-1 reduced bladder tumor cell proliferation and promoted apoptosis. 

In addition, Yan et al. (2015[23]) reported that the lncRNA-PCAT-1 may present as a new biomarker for poor prognosis in hepatocellular carcinoma. However, other investigations proposed that the lncRNA-PCAT-1 gene overexpression was correlated with the clinical characteristics and poor overall survival of hepatocellular carcinoma (HCC) subjects (Zhang et al., 2017[24]; Ren et al., 2017[11]). 

Zhang et al. (2017[24]) revealed that the lncRNA-PCAT-1 stimulated HCC invasion and metastasis by competitively binding to miR-129-5p, which in turn suppressed the expression of high mobility group box 1 (HMGB1). In addition, the lncRNA-PCAT-1 played as a ceRNA against miR-122, and that inhibition of lncRNA-PCAT-1 suppressed the advancement of ECC by decreasing Wnt/β-catenin signaling pathway by miR-122 repression and Wnt group member 1 (WNT1) expression (Zhang et al., 2017[24]).

Zhen et al. (2018[26]) reported that up-regulation of lncRNA-PCAT-1 enhanced the proliferation and growth of esophageal cancer cells and cisplatin chemosensitivity. The genetic procedures regulating the chemoresistance of ESCC remained unknown. The findings showed that up-regulation of lncRNA-PCAT-1 could trigger cell growth and proliferation of esophageal cells. Because of lncRNA-PCAT-1 knockdown leads to suppression of cell growth and proliferation. In addition, lncRNA-PCAT-1 knockdown increased the chemosensitivity of esophageal cells on cisplatin by inhibiting tumor cell expansion and proliferation and promoting tumor cell apoptosis. Findings from *in vivo* xenograft model demonstrated that knockdown of lncRNA-PCAT-1 significantly suppressed tumor expansion and evolution. These data present personal and significant molecular purposes for the investigation and management of ESCC (Zhen et al., 2018[26]). Our results indicate that the lncRNA-PCAT-1 was significantly overexpressed in tumor tissues compared with the adjacent non-cancerous tissues. These findings support the data reported by Shi et al. (2015[15]), in which lncRNA-PCAT-1 was up-regulated in cancerous tissues of ESCC.

The findings declared that the *lncRNA-PCAT-1* overexpression in patients with ESCC was significantly correlated with the overall survival time (Shi et al., 2015[15]). The molecular process of *lncRNA-PCAT-1* has been declared that the *lncRNA-PCAT-1* is straight linked to polycomb repressive complex 2 (PRC2), a methyltransferase which controls histone H3 methylation at lysine 27 (K27) to inhibit transcription and that *lncRNA-PCAT-1* up-regulation contributes to cell proliferation activated in vitro by target of the PRC2 and is correlated with tumor aggressiveness in prostate malignancy (Shi et al., 2015[15]). 

Our investigation is a preliminary observational study that could open to new questions regarding the effect of aberrant expression of *-PCAT-1* on ESCC development of Iranian patients. The practical implication of our findings is that the *lncRNA*-*PCAT-1* might be proposed as a novel candidate gene, which is involved in the development and progression of ESCC. In addition, targeting *lncRNA*-*PCAT-1* molecule may be considered as potential therapeutic element in ESCC. The finding revealed a positive association between up-regulation of the *lncRNA-PCAT-1* and clinical stage and poor prognosis of cancers. However, our results showed that aberrant expression of *lncRNA-PCAT-1* was consistent with the previous report in prostate cancer and ESCC. There are still several limitations in our investigation. The number of sample size is not adequate for subgroup analysis of several clinical features. This small number may have led to the result that *lncRNA-PCAT-1* expression level had no significant impact on lymph node metastasis.

However, their results reinforce a notable appearance of *lncRNA-PCAT-1* in pathogenesis of ESCC and recommends that the cancer-specific functions of this lncRNA in tumorigenesis was assessed in future investigations. In addition, future studies are needed to clarify the clinical significance of the *lncRNA-PCAT-1* expression level in other types of malignancies. In addition, the molecular mechanisms of the *lncRNA-PCAT-1* in tumor progression and development needs to be determined in the next study.

In conclusion, our findings suggested that the *lncRNA-PCAT-1* could be involved in the development and progression of ESCC, as a candidate prognostic biomarker in patients with ESCC to improve clinical management. 

## Acknowledgements

This article was extracted from an MS.c thesis (IR. 910873730) at Ahar Branch Islamic Azad University. We would like to appreciate all the participants. 

## Conflict of interest

The authors declare that they have no conflict of interest.

## Figures and Tables

**Table 1:**
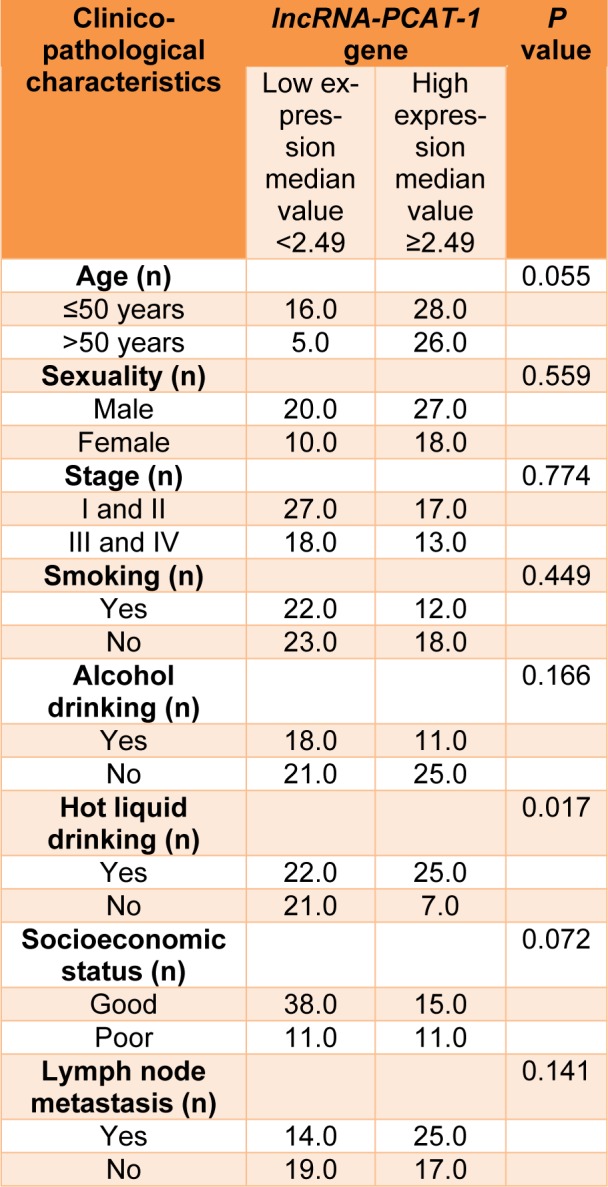
Correlation between *lncRNA-PCAT-1* gene expression and clinicopathological characteristics of ESCC patients.
